# Impact of Tumor Location on the Efficacy of Lateral and Mesenteric Lymph Node Dissection in Patients With Rectal Cancer Treated by Upfront Surgery

**DOI:** 10.1002/ags3.70065

**Published:** 2025-07-10

**Authors:** Tomofumi Uotani, Hiroshi Nagata, Yasuyuki Takamizawa, Konosuke Moritani, Shunsuke Tsukamoto, Tsutomu Fujii, Yukihide Kanemitsu

**Affiliations:** ^1^ Department of Colorectal Surgery National Cancer Center Hospital Tokyo Japan; ^2^ Department of Surgery and Science, Faculty of Medicine, Academic Assembly University of Toyama Toyama Japan

**Keywords:** lymph node excision, lymphatic metastasis, prognosis, rectal neoplasms, treatment outcome

## Abstract

**Background:**

The relationship between tumor location and lymphatic flow is generally known to affect the efficacy of lymph node dissection, but the specific impact in rectal cancer remains unclear. This study investigated the frequency of lymph node metastasis (LNM) and the efficacy of lymph node dissection based on tumor location.

**Methods:**

We retrospectively investigated 882 patients with rectal adenocarcinoma who underwent total mesorectal excision with lateral lymph node dissection. Tumors were categorized by primary site into Ra (from the lower edge of S2 to the peritoneal reflection, *n* = 95), Rb (from the peritoneal reflection to the upper edge of the anal canal, *n* = 713), and P (anal canal, *n* = 74). LNM rates and dissection efficacy were assessed at each station. The therapeutic value index (TVI) was calculated as the LNM rate multiplied by the 5‐year overall survival rate.

**Results:**

LNM was observed in 447 patients (50.7%). Mesenteric LNM rates and the TVI were higher for tumors located more orally (49.5% and 43 for Ra, 46.1% and 29.7 for Rb, 43.2% and 17.6 for P), whereas lateral LNM rates and the TVI were higher for tumors located more anally (7.4% and 3.7 for Ra, 16.3% and 8 for Rb, 29.7% and 14.1 for P). Regardless of tumor location, the TVI in the lateral region was high in the distal internal iliac and obturator areas.

**Conclusions:**

Tumor location influences LNM frequency and lymph node dissection efficacy in rectal cancer. Treatment strategies should be individualized based on tumor location to improve outcomes.

## Graphical abstract

This study examined the impact of tumor location on lymph node metastasis (LNM) patterns and the efficacy of lymph node dissection in rectal cancer. Analyzing 882 patients who underwent total mesorectal excision with lateral lymph node dissection, we found that mesenteric LNM rates were higher in tumors located more orally, whereas lateral LNM rates were higher in tumors located more anally. The therapeutic efficacy of lymph node dissection varied by tumor location, highlighting the need for individualized treatment strategies to optimize oncologic outcomes.

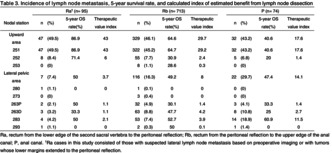

## 1 Introduction

Colorectal cancer is the third most frequently diagnosed cancer globally and ranks second in cancer‐related mortality [1]. Approximately 40% of these cases are rectal cancer. Treatment strategies for locally advanced rectal cancer (LARC) differ by geographic region. In Japan, surgery including lateral lymph node dissection (LLND) remains the standard treatment [2]. In contrast, preoperative radiotherapy or chemoradiotherapy followed by surgery has historically been the standard treatment in Western countries [3, 4]. In recent years, treatment methods have advanced with the development of personalized medicine based on genetic testing [5, 6, 7] and total neoadjuvant therapy [8, 9, 10]. Despite these advances, some patients still have poor outcomes, including early recurrence.

Lateral lymph node metastasis (LLNM) is one of the critical factors affecting the prognosis of LARC [11], and in Japan is considered to be a local disease that is controllable with localized treatment. Differences in disease concepts influence treatment strategies; however, data on the risk of LLNM and the efficacy of treatments for lymph node involvement are important in terms of guiding treatment decisions.

LLND has been developed based on research into the lymphatic flow in the rectum [12, 13]. This is particularly important in LARC located below the peritoneal reflection, where lateral lymphatic flow is considered crucial. In other cancers, the relationship between tumor location and lymphatic flow has been reported to affect LNM patterns and the efficacy of dissection [14, 15]. In rectal cancer, lymphatic flow is multidirectional—upward, lateral, and downward [16]. However, the patterns of LNM and the effectiveness of lymph node dissection based on the precise tumor location remain incompletely understood.

The aim of this study was to identify the frequency of LNM and the efficacy of lymph node dissection at various stations based on tumor location in patients with LARC.

## 2 Methods

### 2.1 Study Population

The study included patients with rectal adenocarcinoma who underwent upfront total mesorectal excision (TME) plus LLND at our institution between January 1975 and December 2017. Stage IV and rectosigmoid cancers were excluded. All patients underwent preoperative lower gastrointestinal endoscopy, computed tomography (CT), endoscopic ultrasound, and/or magnetic resonance imaging.

### 2.2 Definitions of Tumor Location

The Japanese guidelines divide the rectum into three sections: RS, Ra, and Rb [17]. RS is defined as the rectum from the sacral promontory to the lower edge of the second sacral vertebra, Ra as the rectum from the lower edge of the second sacral vertebra to the peritoneal reflection, and Rb as the rectum from the peritoneal reflection to the upper edge of the anal canal. In this study, cases classified as Ra were those in which the inferior margin of the tumor extended beyond the peritoneal reflection while the main tumor site remained within the Ra region. We examined the frequency of LNM and the efficacy of lymph node dissection based on the primary site, categorizing cases into Ra, Rb, and P (the anal canal).

### 2.3 Treatment Strategies for Rectal Cancer

Based on preoperative imaging and intraoperative assessment, patients with clinical T1 or T2 tumors and no signs of LLNM were treated with standard TME alone. In contrast, those with cT3 or cT4 tumors located at or below the peritoneal reflection, or with suspected LLNM underwent additional LLND alongside TME. Neoadjuvant treatment was not routinely administered, in accordance with the guidelines established by the Japanese Society for Cancer of the Colon and Rectum [2], except for patients with high‐risk features for local recurrence, such as a threatened circumferential resection margin or suspected invasion of adjacent organs. Adjuvant chemotherapy was routinely administered for stage III disease and considered for high‐risk stage II cases, although in practice it was rarely given to stage II patients. Postoperative radiotherapy was considered in cases with pathologically positive resection margins.

### 2.4 Extent of Lateral Lymph Node Dissection

The methods and extent of LLND varied over time. Before 1997, LLND was performed en bloc and included the pelvic autonomic nerves. After 1998, nerve‐sparing techniques were used in cases without suspected LLNM. In the early and middle periods (1975–1997), bilateral LLND was performed in most cases. In the later period (1998–2017), bilateral LLND was routinely performed regardless of whether LLNM was present. However, selective unilateral LLND was performed in cases where tumor localization was limited and for patients with high‐risk comorbidities. In accordance with the TNM classification [18], inguinal LNM was considered a regional LNM in cases of anal canal cancer, whereas it was regarded as systemic disease in other rectal cancer. Prophylactic dissection of inguinal lymph nodes was not routinely performed. However, in cases of anal canal cancer with clinically suspected inguinal LNM, selective dissection was undertaken.

Postoperatively, all regional lymph nodes were individually extracted from the adipose tissue in the specimen, and the number and location of these nodes were recorded according to the Japanese General Rules criteria [17]. These criteria classify the lateral pelvic region into the aortic bifurcation area (280), common iliac area (273), proximal internal iliac area (263P), distal internal iliac area (263D), obturator area (283), and external iliac area (293) (Figure S1). All specimens were fixed in formalin and embedded in paraffin. Sections were cut to a width of 4 μm, and hematoxylin–eosin staining was performed for histopathological evaluation. Each lymph node was assessed using a single section.

### 2.5 Postoperative Surveillance

Postoperative follow‐up was conducted every 3 months during the first 2 years and every 6 months for the subsequent 3 years, with tumor markers measured at each visit. Imaging of the liver (by ultrasound or CT) and chest (by radiography or CT) was performed every 3–6 months, with colonoscopy performed every 2–3 years. Follow‐up data were prospectively recorded until the occurrence of an event or the conclusion of the study in December 2023, whichever came first. The median follow‐up period for survivors was 91.5 months (range, 0–369). Local recurrence was defined as recurrence of disease within the pelvic cavity, which could be diagnosed clinically, radiographically, or histologically.

### 2.6 The Therapeutic Value Index

The therapeutic efficacy of lymph node dissection was assessed using previously reported methods [19]. This approach assumed that patients who survived long‐term following lymph node dissection would not have survived if the metastatic lymph nodes had not been removed. The rate of LNM was calculated by dividing the number of patients with metastasis in each station by the number of patients who underwent lymphadenectomy at that station. The 5‐year overall survival (OS) rate of patients with LNM at each station was calculated regardless of metastasis at other stations. The efficacy index of lymph node dissection was determined by multiplying the frequency of metastasis by the 5‐year survival rate of patients with metastasis at that station. Thus, survival benefit was assessed independently of TNM staging. The study was approved by the Institutional Review Board of the National Cancer Center Hospital (IRB code: 2017–437) and performed in accordance with the Declaration of Helsinki. The need for informed consent was waived owing to the retrospective nature of this study.

### 2.7 Statistical Analysis

Continuous variables were compared between groups using the Mann–Whitney *U* test or the Kruskal–Wallis test and categorical variables were compared using the chi‐squared test. Survival curves were generated using the Kaplan–Meier method [20] and compared using the log‐rank test. Given that two pairwise comparisons were performed, the significance level was adjusted using the Bonferroni correction (*α* = 0.05/2 = 0.025) to account for multiple testing. All statistical analyses were performed using EZR software (Saitama Medical Center, Jichi Medical University, Saitama, Japan) [21]. All tests were two‐sided. For comparisons with multiple testing, a *p*‐value < 0.025 was considered statistically significant, while for other analyses, a *p*‐value < 0.05 was used as the significance threshold.

## 3 Results

### 3.1 Patient Characteristics and Treatment Details

A total of 882 patients with rectal adenocarcinoma underwent TME with LLND at our institution. Their clinicopathological and treatment characteristics are summarized in Table 1. There were no significant differences in sex distribution among the Ra (66.3% male), Rb (65.6% male), and P (63.5% male) groups (*p* = 0.922). Median age was 61 years in the Ra group, 58 years in the Rb group, and 59 years in the P group, with no significant difference (*p* = 0.103). Operative time was significantly longer in the P group (median, 381 min) compared to the Ra (320 min) and Rb (334 min) groups (*p* = 0.026). Blood loss was significantly lower in the Ra group (median, 362 mL) compared to the Rb (606 mL) and P (555 mL) groups (*p* = 0.002). Surgical procedures differed significantly according to tumor location (*p* < 0.001). Low anterior resection (LAR) was most common in Ra tumors (72.6%) and was not performed in P tumors. Abdominoperineal resection (APR) was the predominant procedure in P tumors (81.1%) and also common in Rb tumors (42.9%), but rare in Ra tumors (7.4%). Total pelvic exenteration (TPE) was more frequently performed in Ra tumors (17.9%) than in Rb (5.6%) and P tumors (10.8%). Bilateral LLND was performed in a similar proportion of patients across groups (Ra: 82.1%, Rb: 79.5%, P: 79.7%; *p* = 0.841). There were no 30‐day postoperative deaths in the Ra or P groups, while 2 deaths occurred in the Rb group (0.3%). Postoperative chemotherapy was administered more frequently in the Ra (37.9%) and P (33.8%) groups compared to the Rb group (25.0%), though this difference did not reach statistical significance (*p* = 0.104). Postoperative chemoradiotherapy and radiotherapy alone were rarely used across all groups.

**TABLE 1:** Characteristics and treatment details of patients with lateral lymph node dissection.

	Ra^a^ (*n* = 95)	Rb (*n* = 713)	P (*n* = 74)	*p*
Sex				0.922
Male	63 (66.3)	468 (65.6)	47 (63.5)	
Female	32 (33.7)	245 (34.4)	27 (36.5)	
Age (y)^b^	61 (28–80)	58 (19–84)	59 (30–78)	0.103
Operative time, min^b^	320 (158–720)	334 (147–733)	381 (165–815)	0.026
Blood loss, mL^b^	362 (32–2901)	606 (8–6300)	555 (28–5000)	0.002
Operation				< 0.001
APR	7 (7.4)	306 (42.9)	60 (81.1)	
Hartmann	1 (1.1)	7 (1.0)	0 (0.0)	
ISR	0 (0.0)	74 (10.4)	6 (8.1)	
TPE	17 (17.9)	40 (5.6)	8 (10.8)	
LAR	69 (72.6)	282 (39.6)	0 (0.0)	
Total colectomy	1 (1.1)	1 (0.1)	0 (0.0)	
Unknow	0 (0.0)	3 (0.4)	0 (0.0)	
LLND				0.841
Bilateral	78 (82.1)	567 (79.5)	59 (79.7)	
Unilateral	17 (17.9)	146 (20.5)	15 (20.3)	
30‐day postoperative death	0 (0.0)	2 (0.3)	0 (0.0)	
Adjuvant therapy				0.104
Postoperative radiotherapy	0 (0.0)	5 (0.7)	0 (0.0)	
Postoperative chemotherapy	36 (37.9)	178 (25.0)	25 (33.8)	
Postoperative chemoradiotherapy	0 (0.0)	9 (1.3)	2 (2.7)	
None	59 (62.1)	511 (71.7)	46 (62.2)	
Unknown	0 (0.0)	10 (1.4)	1 (1.4)	

*Note:* Values in parentheses are percentages unless otherwise indicated.

Abbreviations: APR, abdominoperineal resection; ISR, intersphincteric resection; LAR, low anterior resection; LLND, lateral lymph node dissection; P, anal canal; Ra, rectum from the lower edge of the second sacral vertebra to the peritoneal reflection; Rb, rectum from the peritoneal reflection to the upper edge of the anal canal; TPE, total pelvic exenteration.

^a^ Ra cases in this study consisted of those with suspected lateral lymph node metastasis based on preoperative imaging or with tumors whose lower margins extended to the peritoneal reflection.

^b^ values are median (range).

### 3.2 Histopathological Findings

The histopathological characteristics of patients by tumor location are presented in Table 2. The median number of lymph nodes examined per patient was significantly higher in Ra tumors (43) compared to Rb (39) and P tumors (34) (*p* = 0.012). However, the number of mesenteric and lateral lymph node metastases per patient did not significantly differ among groups. The median tumor size was larger in Ra tumors (6.5 cm) than in Rb and P tumors (5 cm each) (*p* < 0.001). Poorly differentiated, mucinous, or signet‐ring cell carcinoma was more frequently observed in P tumors (20.3%) than in Ra (6.3%) and Rb tumors (6.0%) (*p* < 0.001). Regarding tumor depth, T4 tumors were more frequent in Ra (15.8%) and P (13.5%) groups than in Rb (5.2%) (*p* = 0.001), while the overall pTNM stage distribution did not significantly differ among groups (*p* = 0.312). Positive radial margin was significantly more frequent in the P group (8.1%) compared to the Ra and Rb groups (both 2.1%) (*p* = 0.008).

**TABLE 2:** Pathological findings in patients undergoing lateral lymph node dissection.

	Ra^a^ (*n* = 95)	Rb (*n* = 713)	P (*n* = 74)	*p*
No. of lymph nodes examined per patient^b^	43 (11–99)	39 (3–98)	34 (8–95)	0.012
No. of mesenteric lymph node metastases per patient^b^	4 (1–37)	3 (1–25)	2 (1–19)	0.415
No. of lateral lymph node metastases per patient^b^	2 (1–5)	1.5 (1–10)	1 (1–4)	0.295
Tumor size, cm*	6.5 (2–18)	5 (2–21)	5 (1–12)	< 0.001
Tumor differentiation				< 0.001
Well or moderate	89 (93.7)	670 (94.0)	59 (79.7)	
Poor or mucinous or signet ring cell	6 (6.3)	43 (6.0)	15 (20.3)	
pT‐stage				0.001
T1	2 (2.1)	20 (2.8)	3 (4.1)	
T2	16 (16.8)	196 (27.5)	16 (21.6)	
T3	62 (65.3)	460 (64.5)	45 (60.8)	
T4	15 (15.8)	37 (5.2)	10 (13.5)	
pTNM stage				0.312
I	12 (12.6)	144 (20.2)	12 (16.2)	
II	35 (36.8)	211 (29.6)	21 (28.4)	
III	48 (50.5)	358 (50.2)	41 (55.4)	
Positive radial margin	2 (2.1)	15 (2.1)	6 (8.1)	0.008

*Note:* Values in parentheses are percentages unless otherwise indicated.

Abbreviations: P, anal canal; Ra, rectum from the lower edge of the second sacral vertebra to the peritoneal reflection; Rb, rectum from the peritoneal reflection to the upper edge of the anal canal.

^a^ Ra cases in this study consisted of those with suspected lateral lymph node metastasis based on preoperative imaging or with tumors whose lower margins extended to the peritoneal reflection.

^b^ values are median (range).

### 3.3 Frequency of Metastasis and Therapeutic Efficacy of Lymph Node Dissection

Patients were categorized based on the primary site as Ra (*n* = 95), Rb (*n* = 713), or P (*n* = 74). Inguinal LNM was observed in five cases of anal canal cancer. The frequency of LNM by tumor site is shown in Table 3. Examination of the frequency of LNM according to mesenteric and lateral pelvic region showed the mesenteric metastasis rate to be 49.5% for Ra, 46.1% for Rb, and 43.2% for P, indicating higher metastasis rates for tumors located closer to the oral side. In contrast, LLNM rates increased when the tumor was located closer to the anal side, with rates of 7.4% for Ra, 16.3% for Rb, and 29.7% for P. In the lateral pelvic regions, the metastasis rates were higher in the 263D and 283 stations, regardless of tumor location, with P tumors showing particularly high rates at 263D (10.8%) and 283 (18.9%).

**TABLE 3:** Incidence of lymph node metastasis, 5‐year survival rate, and calculated index of estimated benefit from lymph node dissection.

Nodal station	Ra^a^ (*n* = 95)			Rb (*n* = 713)			P (*n* = 74)		
	*n* (%)	5‐year OS rate (%)	Therapeutic value index	*n* (%)	5‐year OS rate (%)	Therapeutic value index	*n* (%)	5‐year OS rate (%)	Therapeutic value index
Upward area	47 (49.5)	86.9	43	329 (46.1)	64.6	29.7	32 (43.2)	40.6	17.6
251	47 (49.5)	86.9	43	322 (45.2)	64.7	29.2	32 (43.2)	40.6	17.6
252	8 (8.4)	71.4	6	55 (7.7)	30.9	2.4	5 (6.8)	20	1.4
253	0 (0)			8 (1.1)	28.6	0.3	0 (0)		
Lateral pelvic area	7 (7.4)	50	3.7	116 (16.3)	49.2	8	22 (29.7)	47.4	14.1
280	1 (1.1)	0	0	1 (0.1)	0	0	0 (0)		
273	0 (0)			3 (0.4)	0	0	0 (0)		
263P	2 (2.1)	50	1.1	32 (4.9)	30.1	1.4	3 (4.1)	33.3	1.4
263D	3 (3.2)	33.3	1.1	63 (8.8)	47.7	4.2	8 (10.8)	25	2.7
283	4 (4.2)	50	2.1	53 (7.4)	52.7	3.9	14 (18.9)	60.9	11.5
293	1 (1.1)	0	0	2 (0.3)	50	0.1	1 (1.4)	0	0

Abbreviations: P, anal canal; Ra, rectum from the lower edge of the second sacral vertebra to the peritoneal reflection; Rb, rectum from the peritoneal reflection to the upper edge of the anal canal.

^a^ Ra cases in this study consisted of those with suspected lateral lymph node metastasis based on preoperative imaging or with tumors whose lower margins extended to the peritoneal reflection.

The efficacy of lymph node dissection for mesenteric lymph nodes was higher when the tumors were located closer to the oral side, with a therapeutic value index (TVI) of 43 for Ra, 29.7 for Rb, and 17.6 for P. Conversely, for the lateral pelvic lymph nodes, the TVI was higher for tumors located nearer to the anal side, with an index of 3.7 for Ra, 8 for Rb, and 14.1 for P. Notably, in the lateral pelvic region, regardless of tumor location, the TVI was particularly high for the 263D and 283 stations, with the 283 station in P cases being highest at 11.5.

### 3.4 Prognosis According to Location of LNM

**FIGURE 1:**
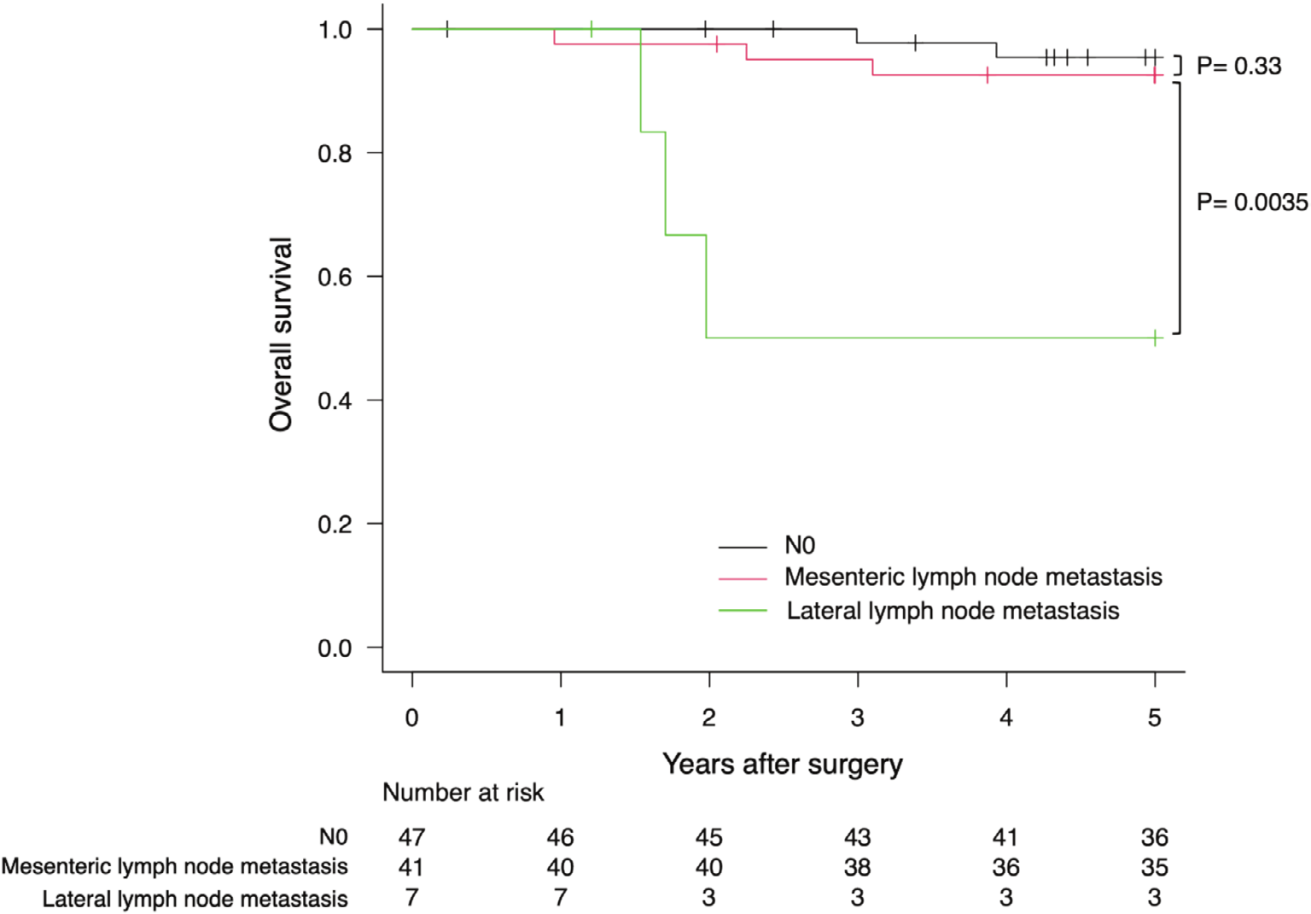
Kaplan–Meier plots of overall survival for tumors with the primary site in Ra.

**FIGURE 2:**
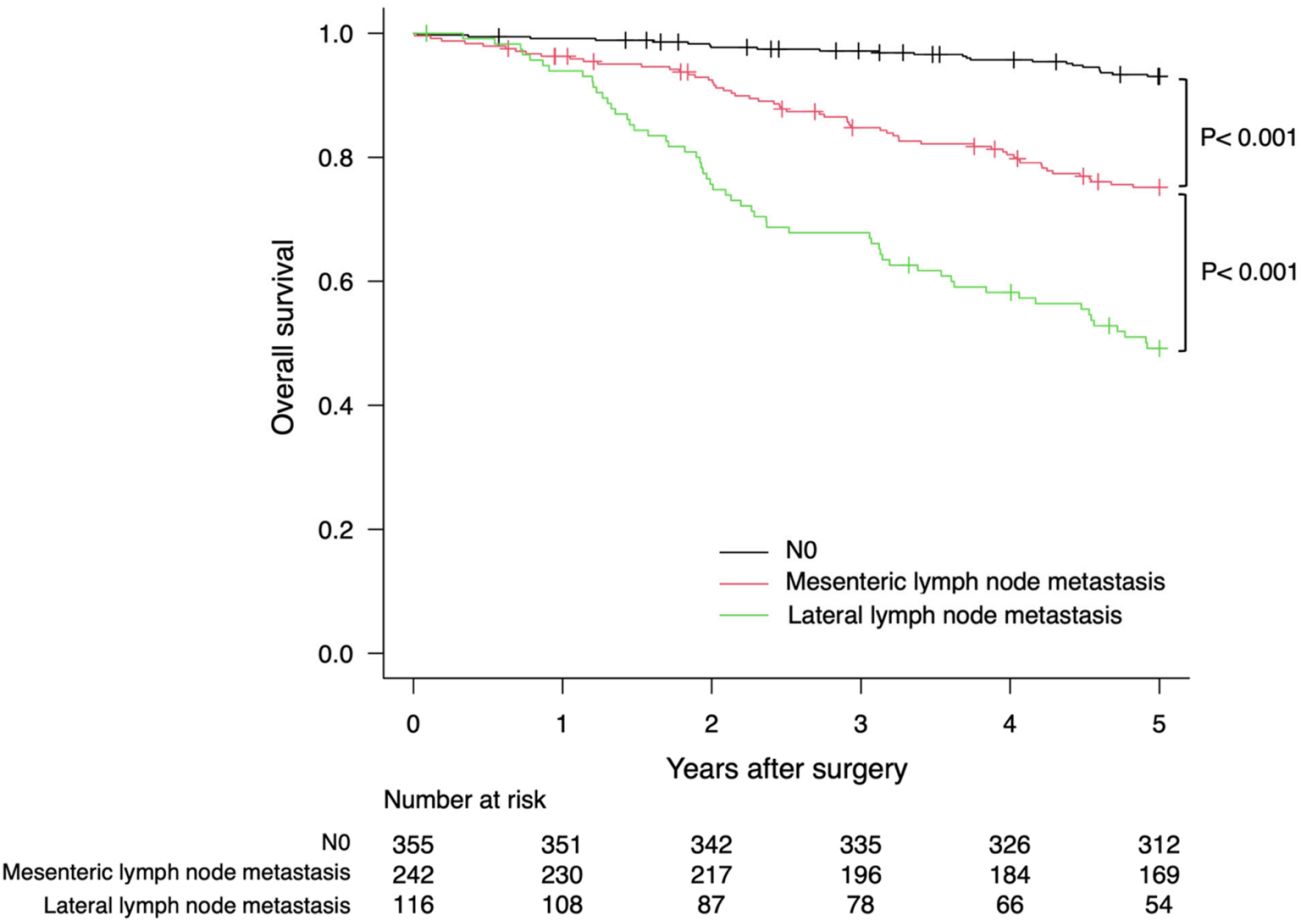
Kaplan–Meier plots of overall survival for tumors with the primary site in Rb.

**FIGURE 3:**
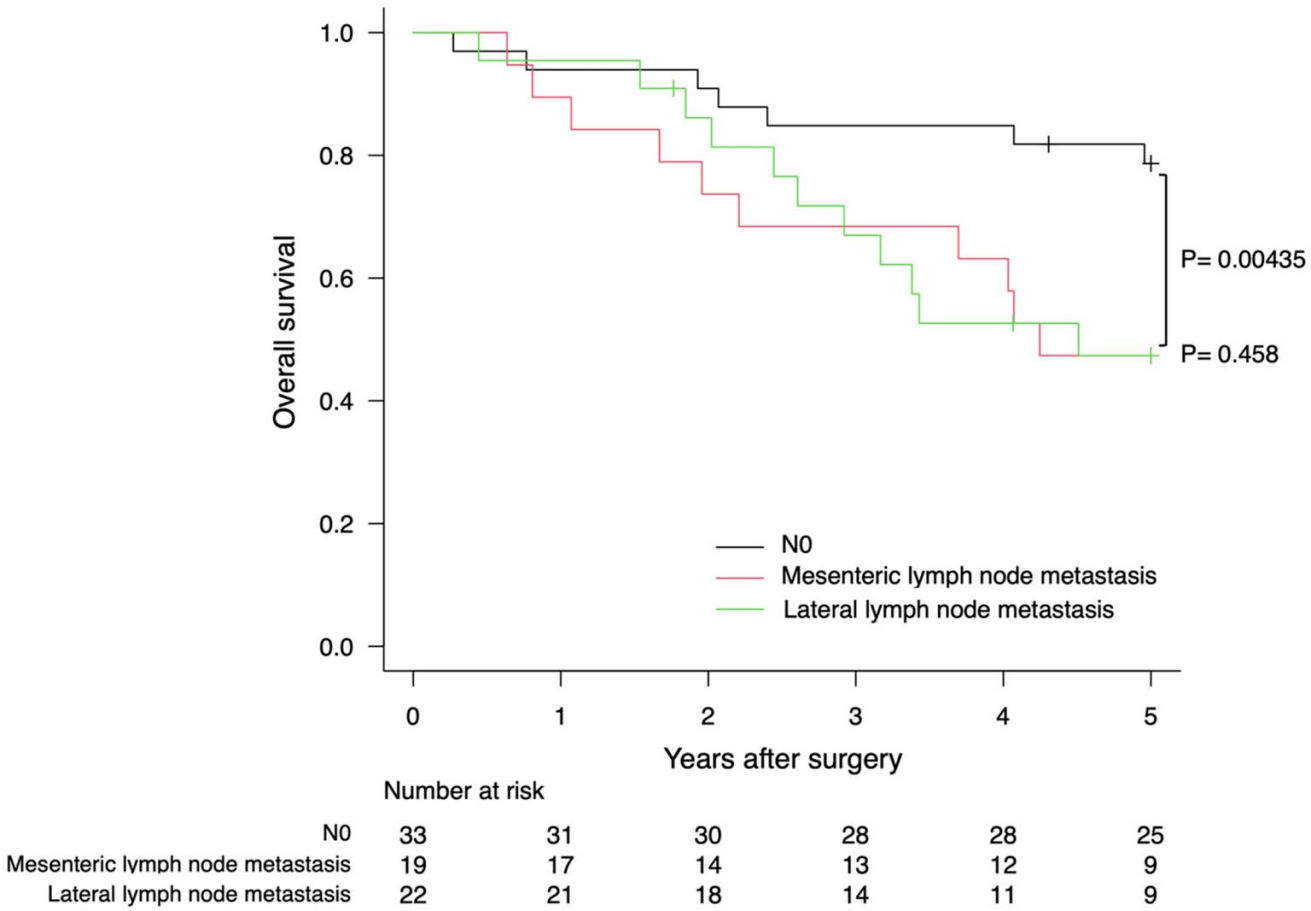
Kaplan–Meier plots of overall survival for tumors with the primary site in P.

Kaplan–Meier curves for OS based on the location of LNM are shown for each tumor site in Figures 1, 2, 3. LLNM was associated with poor prognosis regardless of the tumor site. For mesenteric metastasis, Ra showed no significant difference in prognosis compared to N0. However, Rb had a worse prognosis than N0 but a better prognosis than LLNM.

Recurrence patterns by tumor location are summarized in Table 4. In the overall cohort, the recurrence rate was significantly higher in P tumors (54.1%) compared to Ra (29.5%) and Rb (31.0%) tumors (*p* = 0.001). Distant recurrence was also significantly more frequent in P tumors (41.9%) than in Ra (21.1%) and Rb (23.0%) tumors (*p* = 0.001). Lung metastases occurred most frequently in the P group (25.7%), with a statistically significant difference across groups (*p* = 0.029). No significant differences were observed in local or liver recurrence rates. Among patients with mesenteric lymph node metastases, recurrence occurred in 63.2% of P tumors, 41.5% of Ra tumors, and 36.8% of Rb tumors, though the difference was not statistically significant (*p* = 0.071). Similarly, no significant differences were found in local or distant recurrence patterns among the groups. In patients with lateral lymph node metastases, recurrence rates were uniformly high across all groups: 85.7% in Ra, 67.2% in Rb, and 77.3% in P tumors (*p* = 0.411). Local recurrence occurred in 25.0%–28.6% of cases, and distant recurrence in approximately 50% of cases across all groups, without significant intergroup differences. Subgroup analyses of recurrence patterns in patients who underwent bilateral LLND and those who underwent unilateral LLND are provided in Tables S4 and S5, respectively.

**TABLE 4:** Recurrence rates and patterns according to tumor location.

	All patients				Patients with mesenteric lymph node metastases				Patients with lateral lymph node metastases			
	Ra^a^ (*n* = 95)	Rb (*n* = 713)	P (*n* = 74)	*p*	Ra^a^ (*n* = 41)	Rb (*n* = 242)	P (*n* = 19)	*p*	Ra^a^ (*n* = 7)	Rb (*n* = 116)	P (*n* = 22)	*p*
Recurrence (%)	28 (29.5)	221 (31.0)	40 (54.1)	0.001	17 (41.5)	89 (36.8)	12 (63.2)	0.071	6 (85.7)	78 (67.2)	17 (77.3)	0.411
Local (%)	8 (8.4)	57 (8.0)	9 (12.2)	0.469	4 (9.8)	21 (8.7)	2 (10.5)	0.792	2 (28.6)	29 (25.0)	6 (27.3)	0.958
Distant (%)	20 (21.1)	164 (23.0)	31 (41.9)	0.001	13 (31.7)	68 (28.1)	10 (52.6)	0.083	4 (57.1)	49 (42.2)	11 (50.0)	0.62
Liver (%)	7 (7.4)	62 (8.7)	5 (6.8)	0.79	4 (9.8)	18 (7.4)	2 (10.5)	0.649	3 (42.9)	21 (18.1)	1 (4.5)	0.056
Lung (%)	11 (11.6)	107 (15.0)	19 (25.7)	0.029	7 (17.1)	54 (22.3)	4 (21.1)	0.773	1 (14.3)	29 (25.0)	8 (36.4)	0.412

Abbreviations: P, anal canal; Ra, rectum from the lower edge of the second sacral vertebra to the peritoneal reflection; Rb, rectum from the peritoneal reflection to the upper edge of the anal canal.

^a^ Ra cases in this study consisted of those with suspected lateral lymph node metastasis based on preoperative imaging or with tumors whose lower margins extended to the peritoneal reflection.

## 4 Discussion

This study investigated the impact of tumor location on the frequency of LNM and the therapeutic efficacy of lymph node dissection in patients with rectal cancer treated by upfront surgery. Its findings indicated that the therapeutic efficacy of mesenteric lymph node dissection was greater for tumors located closer to the oral side, whereas the efficacy of LLND was greater for tumors located closer to the anal side. Moreover, tumors primarily located in the anal canal (i.e., P tumors) were associated with poor prognosis, even when metastasis was limited to the mesenteric lymph nodes.

Furthermore, because the study covered a long‐time span (1975–2017), we also analyzed OS and relapse‐free survival (RFS) before and after the routine introduction of nerve‐sparing LLND in 1998 (Figures S2 and S3). Although OS improved significantly in the later period, RFS remained comparable between the two periods. This suggests that the observed improvement in OS may largely reflect broader advancements in perioperative care, surgical techniques, and supportive therapies, rather than the cancer‐specific effects of LLND itself. Importantly, although the TVI is calculated using OS, it specifically reflects the survival of patients with LNM at each station, providing a relative measure of dissection efficacy across tumor locations. Therefore, the main comparative findings of this study—focused on anatomical patterns of metastasis and the relative efficacy of lymph node dissection by tumor location—are considered to remain valid despite era‐related improvements in general medical care.

To date, only a few studies have systematically evaluated the association between tumor location and LNM patterns in rectal cancer. Takahashi et al. [22] reported a high rate of LLNM (16.4%) in rectal cancers located within 5 cm of the dentate line. They also showed that tumors located closer to the anus were more likely to develop lateral metastasis. Similarly, Steup et al. [23] found a higher incidence of LLNM in Rb rectal cancers than in Ra rectal cancers. While these findings are consistent with our results, neither of those studies assessed the therapeutic efficacy of lymph node dissection.

In this study, the efficacy of lymph node dissection was assessed according to tumor location. Mesenteric lymph node dissection was most effective for tumors closer to the oral side, whereas LLND was most effective for tumors closer to the anal side. Previous studies on other types of cancer have suggested that the relationship between tumor location and lymphatic flow may influence both the frequency of metastasis and therapeutic efficacy of lymph node dissection [14, 15]. Our findings are consistent with this notion, likely reflecting differences in the anatomical relationship between tumor location and lymphatic drainage. An analysis of the therapeutic efficacy of lymph node dissection by lymph node station revealed consistently high efficacy in the distal internal iliac region (263D) and the obturator region (283), regardless of tumor location. Ueno et al. [24] further supported the importance of this area by demonstrating that the TVI was particularly high when including not only these regions but also the proximal internal iliac region as part of the so‐called “vulnerable field,” which accounted for the majority of effective lateral dissections. Such anatomical areas have also been highlighted in other reports, identifying these lateral regions as key targets of LLND to achieve oncologic benefit [25, 26].

Furthermore, our analysis of LNM sites and prognosis by tumor location revealed prognostic differences even among metastases within the same lymph node region. For Ra tumors, the prognosis was negatively impacted predominantly in cases with LLNM. In contrast, the prognosis was significantly worse for patients with both mesenteric and lateral lymph node metastases of Rb and P tumors than in N0 cases. Notably, P tumors with mesenteric LNM had a prognosis comparable with that of those with LLNM. This finding suggests that metastasis to upstream lymph nodes, which are typically less likely to develop metastasis, may reflect a more advanced disease state or a more aggressive tumor behavior. This interpretation is supported by a large multicenter study by Inoue et al. [27] which demonstrated that metastasis to the 253 lymph nodes—located at the root of the inferior mesenteric artery—was associated with extremely poor prognosis and a low TVI, especially when coexisting with lateral pelvic LNM. Such findings highlight the potential of tumor location and LNM to be valuable indicators for identifying cases with a poor prognosis and refining risk stratification strategies.

This study has several limitations. First, the study covers a period of several decades, during which significant advances in surgical techniques, perioperative management, and adjuvant treatments occurred. However, the standard treatment in Japan, which involves upfront surgery, remained unchanged throughout this period, reducing the impact of treatment variations. Second, the study had a retrospective design and was performed at a single center, which may have introduced institution‐specific biases or variations in treatment approaches. Therefore, its findings may not be fully generalizable to other institutions, particularly those in Western countries where LLND is not routinely performed. Third, there was a degree of selection bias because patients who underwent preoperative treatments were excluded, which meant that the impact of these preoperative therapies on LNM patterns and the prognosis could not be assessed. Moreover, these exclusions may limit the applicability of our findings to patients who receive neoadjuvant therapies, which can alter tumor characteristics, lymphatic drainage, and overall treatment outcomes. Fourth, this study did not account for other potential prognostic factors, such as patient background characteristics, genetic status of colorectal cancer, postoperative complications, or adjuvant treatments. These factors can significantly impact the prognosis, and their inclusion in future studies would provide a more comprehensive understanding of the factors influencing patient outcomes. Finally, due to limitations in the available data, including the absence of specific documentation on the side of recurrence, we were unable to analyze detailed patterns such as whether recurrence occurred on the dissected or contralateral side following selective LLND. We consider this an important area for future research and plan to incorporate more detailed recurrence mapping in subsequent investigations.

Nevertheless, this study clarified how tumor location affects the frequency of LNM and the therapeutic efficacy of lymph node dissection in rectal cancer. Notably, tumors involving the anal canal may have a poor prognosis, even when metastasis is confined to mesenteric lymph nodes. While further investigation is needed, it may be recommended that tumors located closer to the anus, particularly those with mesenteric LNM, receive more intensive treatment, including neoadjuvant therapies, to improve outcomes.

## Author Contributions

**Tomofumi Uotani:** conceptualization, methodology, software, data curation, investigation, writing – original draft, writing – review and editing. **Hiroshi Nagata:** writing – review and editing. **Yasuyuki Takamizawa:** writing – review and editing. **Konosuke Moritani:** writing – review and editing. **Shunsuke Tsukamoto:** writing – review and editing. **Tsutomu Fujii:** writing – review and editing. **Yukihide Kanemitsu:** writing – review and editing, supervision, project administration, methodology, conceptualization.

## Ethics Statement

The study was approved by the Institutional Review Board of the National Cancer Center Hospital (IRB code: 2017–437).

### Consent

The authors have nothing to report.

### Conflicts of Interest

T.F. is an editorial board member of the *Annals of Gastroenterological Surgery*. The remaining authors declare no conflicts of interest.

## Supporting information


Data S1.
