# Supplementary material for: Impact of Tumor Location on the Efficacy of Lateral and Mesenteric Lymph Node Dissection in Patients With Rectal Cancer Treated by Upfront Surgery
Source: Ann Gastroenterol Surg. 2025 Jul 10;9(6):1233–42. doi: 10.1002/ags3.70065 (PMC12586942; doi:10.1002/ags3.70065)
Supplement: Supplementary file 1 — Data S1. [file AGS3-9-1233-s001.docx]

**Supporting Information for**

**Impact of Tumor Location on the Efficacy of Lateral and Mesenteric Lymph Node Dissection in Patients with Rectal Cancer Treated by Upfront Surgery**

Tomofumi Uotani, MD, Hiroshi Nagata MD, PhD, Yasuhiro Takamizawa MD, PhD, Konosuke Moritani, MD, PhD, Shunsuke Tsukamoto, MD, PhD, Tsutomu Fujii, MD, PhD, Yukihide Kanemitsu, MD

**Contents**

Supplementary Figure S1-3 and Tables S4-5.

**Supplementary Figure S1**. Lymph node groups and station numbers. Red, pericolic/perirectal lymph nodes; blue, intermediate lymph nodes; yellow, main lymph nodes; green, lateral lymph nodes

**Supplementary Figure S2.** Kaplan–Meier plots of overall survival before and after 1998.

**Supplementary Figure S3.** Kaplan–Meier plots of relapse-free survival before and after 1998.

**Supplementary Table S4. Recurrence rates and patterns according to tumor location in patients undergoing bilateral lateral lymph node dissection.**

|  | All patients | | | | | | |  | Patients with mesenteric lymph node metastases | | | | | | |  | Patients with lateral lymph node metastases | | | | | | |
| --- | --- | --- | --- | --- | --- | --- | --- | --- | --- | --- | --- | --- | --- | --- | --- | --- | --- | --- | --- | --- | --- | --- | --- |
|  | Ra^†^  (n= 78) | | Rb  (n= 567) | | P  (n= 59) | | *p*  value |  | Ra^†^  (n= 31) | | Rb  (n= 197) | | P  (n= 13) | | *P*  value |  | Ra^†^  (n= 6) | | Rb  (n= 102) | | P  (n= 19) | | *p*  value |
| Recurrence(%) | 21 | (26.9) | 185 | (32.6) | 32 | (54.2) | 0.001 |  | 12 | (38.7) | 72 | (36.5) | 8 | (61.5) | 0.199 |  | 5 | (83.3) | 70 | (68.6) | 14 | (73.7) | 0.697 |
| Local(%) | 6 | (7.7) | 44 | (7.8) | 7 | (11.9) | 0.541 |  | 3 | (9.7) | 13 | (6.6) | 2 | (15.4) | 0.446 |  | 2 | (33.3) | 27 | (26.5) | 4 | (21.1) | 0.81 |
| Distant(%) | 15 | (19.2) | 141 | (24.9) | 25 | (42.4) | 0.005 |  | 9 | (29.0) | 59 | (29.9) | 6 | (46.2) | 0.46 |  | 3 | (50.0) | 43 | (42.2) | 10 | (52.6) | 0.67 |
| Liver(%) | 5 | ( 6.4) | 52 | (9.2) | 5 | (8.5) | 0.719 |  | 3 | (9.7) | 15 | (7.6) | 2 | (15.4) | 0.59 |  | 2 | (33.3) | 18 | (17.6) | 1 | (5.3) | 0.216 |
| Lung(%) | 8 | (10.3) | 94 | (16.6) | 16 | (27.1) | 0.031 |  | 4 | (12.9) | 47 | (23.9) | 2 | (15.4) | 0.329 |  | 1 | (16.7) | 27 | (26.5) | 7 | (36.8) | 0.539 |

Data in this table are based exclusively on patients who underwent bilateral lateral lymph node dissection.

Ra, rectum from the lower edge of the second sacral vertebra to the peritoneal reflection; Rb, rectum from the peritoneal reflection to the upper edge of the anal canal; P, anal canal. ^†^Ra cases in this study consisted of those with suspected lateral lymph node metastasis based on preoperative imaging or with tumors whose lower margins extended to the peritoneal reflection.

**Supplementary Table S5. Recurrence rates and patterns according to tumor location in patients undergoing unilateral lateral lymph node dissection.**

|  | All patients | | | | | | |  | Patients with mesenteric lymph node metastases | | | | | | |  | Patients with lateral lymph node metastases | | | | | | |
| --- | --- | --- | --- | --- | --- | --- | --- | --- | --- | --- | --- | --- | --- | --- | --- | --- | --- | --- | --- | --- | --- | --- | --- |
|  | Ra^†^  (n= 17) | | Rb  (n= 146) | | P  (n= 15) | | *p*  value |  | Ra^†^  (n= 10) | | Rb  (n= 45) | | P  (n= 6) | | *P*  value |  | Ra^†^  (n= 1) | | Rb  (n= 14) | | P  (n= 3) | | *p*  value |
| Recurrence(%) | 7 | (41.2) | 36 | (24.7) | 8 | (53.3) | 0.032 |  | 5 | (50.0) | 17 | (37.8) | 4 | (66.7) | 0.355 |  | 1 | (100.0) | 8 | (57.1) | 3 | (100.0) | 0.276 |
| Local(%) | 2 | (11.8) | 13 | (8.9) | 2 | (13.3) | 0.812 |  | 1 | (10.0) | 8 | (17.8) | 0 | (0.0) | 0.462 |  | 0 | (0.0) | 2 | (14.3) | 2 | (66.7) | 0.121 |
| Distant(%) | 5 | (29.4) | 23 | (15.8) | 6 | (40.0) | 0.039 |  | 4 | (40.0) | 9 | (20.0) | 4 | (66.7) | 0.037 |  | 1 | (100.0) | 6 | (42.9) | 1 | (33.3) | 0.493 |
| Liver(%) | 2 | (11.8) | 10 | (6.8) | 0 | (0.0) | 0.413 |  | 1 | (10.0) | 3 | (6.7) | 0 | (0.0) | 0.735 |  | 1 | (100.0) | 3 | (21.4) | 0 | (0.0) | 0.113 |
| Lung(%) | 3 | (17.6) | 13 | (8.9) | 3 | (20.0) | 0.257 |  | 3 | (30.0) | 7 | (15.6) | 2 | (33.3) | 0.393 |  | 0 | (0.0) | 2 | (14.3) | 1 | (33.3) | 0.651 |

Data in this table are based exclusively on patients who underwent unilateral lateral lymph node dissection.

Ra, rectum from the lower edge of the second sacral vertebra to the peritoneal reflection; Rb, rectum from the peritoneal reflection to the upper edge of the anal canal; P, anal canal. ^†^Ra cases in this study consisted of those with suspected lateral lymph node metastasis based on preoperative imaging or with tumors whose lower margins extended to the peritoneal reflection.
